# Sexual Violence and Substance Use among First-Year University Women: Differences by Sexual Minority Status

**DOI:** 10.3390/ijerph191610100

**Published:** 2022-08-16

**Authors:** Kenneth W. Griffin, Lisa L. Lindley, Elaine Cooper Russell, Tori Mudd, Christopher Williams, Gilbert J. Botvin

**Affiliations:** 1 Department of Global and Community Health, George Mason University, Fairfax, VA 22030, USA; 2 National Health Promotion Associates, White Plains, NY 10604, USA; 3 Department of Psychology, Purchase College, State University of New York, Purchase, NY 10577, USA; 4 Department of Population Health Sciences, Weill Cornell Medical College, New York, NY 10065, USA

**Keywords:** sexual violence, sexual harassment, sexual assault, substance use, sexual minority, women, university

## Abstract

Sexual violence and substance use are important public health problems among university students. The present study examined rates of sexual violence victimization, perpetration, and substance use among first-year university women. Participants (*n* = 974) attending 14 universities across the United States completed an online confidential survey at the beginning and again later in their first year. The sample included women who identified as heterosexual, bisexual, lesbian, and asexual or questioning. The mean age was 19.1 years and 71.4% were White. Rates of victimization involving sexual harassment and sexual acts without consent were higher among sexual minority women relative to heterosexual women, with bisexual women being most likely to report these outcomes. Compared to heterosexual women, sexual minority women reported more frequent cigarette smoking, marijuana use and intoxication, use of club drugs, and overall illicit drug use. Across sexual violence and substance use outcomes, bisexual women reported the highest rates. Sexual minority women reported more accurate beliefs about sexual violence and consent relative to heterosexual women. Over the course of the first year, bisexual women and those who used illicit substances were more likely to report new incidents of sexual violence victimization. Implications for prevention of sexual violence among women, including sexual minorities, are discussed.

## 1. Introduction

Sexual violence is a serious public health problem among women attending university. University women experience sexual violence at rates that are substantially higher than their male counterparts. In 2020, approximately one in every four undergraduate women in the United States (US) experienced rape or sexual assault through physical force, violence, or incapacitation (26.4%), compared to 6.8% of undergraduate men [1]. Alcohol and substance use are also prevalent among women attending university. According to a 2019 Substance Use and Mental Health Services Administration (SAMHSA) report [2], more than half (53.4%) of full-time university women in the US aged 18–22 currently used alcohol (in the past month), nearly one third (31.3%) engaged in binge drinking, and one in five (19.9%) currently used marijuana. Marijuana use has been increasing among students overall, and this escalation has been more pronounced among university women [3]. The SAMHSA report [2] also indicated that over one in five (21.3%) full-time university women in the US aged 18–22 reported current illicit substance use, which included marijuana, cocaine, heroin, hallucinogens, inhalants, methamphetamines, and misuse of psychotherapeutics and opioids.

Alcohol and substance use increase the risk for sexual violence among university women. Alcohol use is linked to sexual risk taking, and university women under the influence of alcohol have lower inhibitions and are more likely to engage in sexual activities, including “hooking-up” with someone they just met [4]. Heavy drinking and the frequency of hooking-up increase the likelihood that university women will experience sexual violence victimization [5,6]. Marijuana use is another risk factor for sexual violence victimization, and students who use marijuana often do so simultaneously with alcohol misuse [7]. Perpetrators taking advantage of incapacitated victims is the most frequently reported method of sexual assault. Among university women who reported at least one incident of sexual assault, more than half occurred while the victim was incapacitated due to alcohol or drug use [8,9]. 

Existing research shows that other factors are associated with sexual violence and substance use among university women. One such factor is that the first year of university is a high-risk period of transition, particularly among women [10,11,12]. Risk may be higher among first-year women as they embrace new freedoms that typically coincide with college attendance, including entering new social groups and social settings where alcohol use may be prevalent [13]. Sexual assault victimization among university women peaks in the first year, and rates decline in subsequent years [10,11]. Studies have shown that sexual minority women are also at elevated risk for sexual violence victimization and substance use. Sexual minority university women (female students who identify as lesbian, gay, bisexual, or another non-heterosexual identity) experience higher rates of sexual violence compared to their heterosexual counterparts and are far more likely to experience repeat victimization [14,15]. Sexual orientation has been associated with university women’s patterns of alcohol consumption, binge drinking, and increased risk for negative alcohol-related outcomes, including alcohol misuse disorder [16,17,18]. 

Although there is comprehensive extant literature on behavioral health outcomes for emerging adults in college, relatively little is known about the correlates and predictors of sexual violence against women who identify as sexual minorities, particularly during their first year of college. To our knowledge, no studies have directly examined sexual violence among lesbian, bisexual, and asexual/questioning university women and the ways in which sexual harassment and assault may be impacted by prevalent forms of alcohol and drug misuse. The goal of the present study was to examine sexual violence victimization and perpetration, as well as alcohol and substance use among first-year university women. We examined differences between heterosexual and sexual minority women on these outcomes, as well as differences among subgroups of sexual minority women (lesbian/gay, bisexual, and asexual/questioning women). We also examined differences in sexual violence beliefs, consent beliefs, and factors associated with new incidences of sexual violence and substance use over the course of the first year of college among this sample. The main hypotheses guiding this study were informed by minority stress theory [19] which posits that health disparities related to sexual orientation reflect the persistent stigma, prejudice, and discrimination, and resulting hostile and stressful social environments, that many sexual minorities face. By extension, we hypothesized that women who identified as sexual minorities would be at increased risk for sexual violence victimization relative to their heterosexual counterparts. It was also hypothesized that sexual minority status would be associated with heightened risk for problem drug and alcohol use. 

## 2. Materials and Methods

### 2.1. Participants

Participants were invited by email to complete a 30-min online survey assessing measures of health-related knowledge, skills, attitudes, and behaviors at the beginning of their first year of university and again several months later in the same academic year. Approximately 75% of respondents invited to participate in this study completed both online surveys. Prior to completing the surveys, students were asked to read and provide consent to participate. Participants were informed through the consent form that individual identification codes would be used instead of names to ensure confidentiality, allowing for their survey responses to be matched over time. The consent form explained that the survey results would be seen only by project staff, only general information (e.g., group-level statistics) would be used in future reports, and that no individual results would be made available to anyone outside of the research staff. As an added layer of protection, the consent form described that the surveys used Secure Socket Layer (SSL) encryption, the same level of protection used for credit card transactions. The consent form indicated that participation in this project was completely voluntary, and that respondents could refuse to answer any question that made them uncomfortable and were free to withdraw from the study at any time by discontinuing completion of the survey. The study protocol was reviewed and approved by an authorized Institutional Review Board prior to its start. A referral protocol that provided a free and confidential 24-h hotline was available for any students who might experience emotional distress. No participating students reported distress and the protocol was not activated. 

The sample (*n* = 974) consisted of women attending 14 colleges or universities in the United States. Inclusion criteria were women aged 18 or older and matriculating in their first year of university. Approximately 80% of the sample was 18 or 19 years of age with a mean of 19.1 years of age (SD = 2.67, range 18–29). The racial composition of the sample was 71.4% White, 15.3% Black, 7.4% Asian, 2.1% American Indian/Alaska Native, and 1% Native Hawaiian or Other Pacific Islander. Approximately 2.8% reported they were from another race category, and 16.9% of the sample reported that they were of Hispanic ethnicity. 

### 2.2. Measures

*Demographic information.* Data concerning the demographic characteristics of the participants were collected using standard survey items assessing age, race/ethnicity, and gender. Sexual orientation was asked with the question “Which is your sexual orientation?”, with response options of heterosexual, gay/lesbian, bisexual, not sure, and other. There were 18 individuals who selected “other” and wrote in responses, and these were recoded into one of the existing categories (heterosexual, lesbian, bisexual, or asexual/questioning).

*Sexual Harassment.* Sexual violence was assessed with questions asking about sexual harassment and sexual acts without consent, using items adapted from Cantor [20]. To determine if a participant had been the victim of sexual harassment, the survey asked respondents to indicate how often (1) someone made sexual remarks, told jokes or stories that you found uncomfortable or offensive; and (2) someone emailed, texted, phoned, or messaged sexual remarks, jokes, stories, pictures, or videos to you that made you uncomfortable or you found offensive. To assess if a participant had been the perpetrator of sexual harassment, they were asked the same set of questions regarding how often they had perpetrated the acts outlined above (e.g., you made remarks, told jokes or stories that others found uncomfortable or offensive). Response options for the sexual harassment items were “Never” (1); “Yes, More than a Year Ago (2); “Yes, in the Past Year (3); “Yes, in the Past Month (4); and “Yes, in the Past Week (5). Variables were constructed to assess prevalence rates over the participant’s lifetime (ever), within the past year, and within the past month.

*Sexual Acts without Consent.* To determine if a participant had experienced sexual acts without consent, the survey asked respondents to indicate how often (1) someone kissed or sexually touched you without your consent; (2) someone initiated contact with you involving penetration or oral sex without your consent; and (3) you had sexual intercourse when you did not want to because you were too intoxicated to resist. To determine if a participant had been the perpetrator of sexual acts without consent, they were asked the same set of questions regarding how often they perpetrated the acts outlined above (e.g., you kissed or sexually touched someone without their consent). Response options were the same as for sexual harassment.

*Substance Use.* Substance use was assessed with a series of items assessing the frequency of cigarette smoking and the use of alcohol, marijuana, and inhalants [21], with response options ranging from “Never” (1) to “More than once a day” (9). An example includes: About how often (if ever) do you smoke marijuana (weed, pot)? In addition to assessing the frequency of alcohol use and drunkenness using the same frequency response options, alcohol misuse was assessed by asking how many drinks containing alcohol do you have on a typical day when drinking, the largest number of drinks of alcohol on any one occasion during the past month, and how many times were you unable to remember what happened the night before because you had been drinking. The frequency of prescription drug misuse was assessed for stimulants, sedatives, and pain relievers. The frequency of illicit drug use was assessed for cocaine, heroin, phencyclidine (PCP), methamphetamines, club drugs, and an item asked about the frequency of misusing over-the-counter cough medicine. The substance use items are similar to those used in national surveys in the US, such as the Monitoring the Future study [22]. 

*Sexual Assault Beliefs*. Sexual assault beliefs were measured using seven items (α = 0.75), with response options on a 5-point Likert-type scale from “Strongly Disagree” to “Strongly Agree”: (1) Most sexual assaults are committed by strangers; (2) Most sexual assaults occur in dangerous places; (3) If a person doesn’t want to be sexually assaulted, they shouldn’t dress provocatively or act in a flirtatious way; (4) If someone didn’t report it to the police right away, they probably were not sexually assaulted; (5) Victims often make up lies about being sexually assaulted; (6) A person cannot be sexually assaulted by a boyfriend, girlfriend, or spouse; and (7) If a person decides to go with someone to their bedroom, that implies consent to have sex. A summary score of sexual assault beliefs was constructed as the number of statements that an individual agreed with (higher scores represent more inaccurate beliefs).

*Sexual Consent Attitudes.* Consent attitudes were measured with four items (α = 0.60) from a scale by Humphreys and Brousseau [23], with a similar 5-point Likert-type scale: (1) Sometimes, it is okay not to ask for consent; (2) If consent is asked for at the beginning of a sexual encounter, it is not necessary to ask for consent at every step of the sexual interaction; (3) Obtaining sexual consent is more important in a new relationship than in a long-term relationship; and (4) Sexual intercourse is the only sexual activity that requires explicit verbal consent. A summary score of the consent attitudes was constructed as the number of statements to which a respondent agreed (higher scores represent more inaccurate beliefs).

### 2.3. Data Analysis

Bivariate and multivariate statistical methods were used to analyze the data, including *t*-tests, chi-squares, and hierarchical regressions. First, data were checked for errors, response inconsistencies, outliers, and missing data, following standardized data-screening protocols. In the data analyses, the study predictors included sexual minority status, sexual assault beliefs, and sexual consent attitudes. Behavioral outcomes in the analyses included sexual violence victimization and perpetration, focusing on sexual harassment and sexual acts without consent, and substance use. Covariates in the data analyses included race/ethnicity. Differences between groups on the behavioral outcomes were examined with chi-square and one-way analysis of variance tests with Bonferroni post-hoc comparisons. Predictors of the behavioral outcomes over time were examined using logistic regressions, with the Time 2 outcome as the dependent variable and Time 1 outcome as a covariate, along with sexual minority status as a predictor and racial minority status as a covariate.

## 3. Results

### 3.1. Sexual Violence Outcomes

As shown in Table 1, chi-square analyses were used to examine whether sexual minority women differed from heterosexual women on the sexual violence outcomes. Overall, a greater proportion of sexual minority women reported sexual harassment victimization (lifetime, past year, and past month) relative to heterosexual women. For example, past year rates of sexual harassment victimization were 73.8% for sexual minority women vs. 59.9% for heterosexual women (χ^2^(1) = 9.82, *p* < 0.001). Overall, a greater proportion of sexual minority women reported sexual activity without consent victimization (lifetime, past year) relative to heterosexual women. For example, the past year rates of sexual activity without consent victimization were 23.4% for sexual minority women vs. 14.3% for heterosexual women (χ^2^(1) = 7.61, *p* < 0.01). Rates of lifetime sexual harassment perpetration were higher among sexual minority women (22.2%) compared to heterosexual women (13.1%) (χ^2^(1) = 7.76, *p* < 0.01). Rates of perpetration of sexual activity without consent were very low in the sample and no subgroup differences were observed.

One-way ANOVA with Bonferroni post-hoc comparisons revealed that all differences between sexual minority and heterosexual women were due to higher rates among bisexual women. When compared to heterosexual women, bisexual women were significantly more likely to report lifetime, past year, and past month sexual harassment victimization, lifetime and past year sexual activity without consent victimization, and lifetime sexual harassment perpetration.

### 3.2. Substance Use Outcomes

A series of chi-square analyses were used to examine whether sexual minority women differed from heterosexual women on substance use outcomes. Overall, as shown in Table 2, there were no significant differences across groups in terms of overall substance use or prescription drug misuse. For example, approximately half of sexual minority women and heterosexual women reported past month substance use. However, differences were observed for illicit drug use. Overall, a greater proportion of sexual minority women reported illicit drug use (lifetime, past month) relative to heterosexual women. For example, the past month rates of illicit drug use were 27.7% for sexual minority women vs. 17.3% for heterosexual women (χ^2^(1) = 8.50, *p* < 0.01). 

An analysis of individual substances revealed that sexual minority women smoked cigarettes more frequently (M = 1.44, SD = 1.19) than heterosexual women (M = 1.21, SD = 0.86) (t(166.1) = 2.71, *p* = 0.032); sexual minority women smoked marijuana more frequently (M = 2.63, SD = 2.31) than heterosexual women (M = 2.11, SD = 1.97) (t(176.1) = 2.53, *p* = 0.006); sexual minority women smoked marijuana until high more frequently (M = 2.50, SD = 1.94) than heterosexual women (M = 2.03, SD = 1.94) (t(177.9) = 2.37, *p* = 0.019); and sexual minority women took club drugs more frequently (M = 1.14, SD = 00.61) than heterosexual women (M = 1.02, SD = 0.20) (t(145.2) = 2.27, *p* = 0.025). 

One-way ANOVA with Bonferroni post-hoc comparisons revealed that all differences between sexual minority and heterosexual women were due to higher rates among bisexual women relative to heterosexual women. When compared to heterosexual women, bisexual women reported significantly more frequent cigarette smoking, marijuana use and intoxication, and club drug use.

### 3.3. Alcohol Use Outcomes

A series of chi-square analyses were used to examine whether sexual minority women differed from heterosexual women on alcohol use outcomes. Overall, as shown in Table 3, there were no significant differences across groups in terms of lifetime, past year, or past month alcohol use or alcohol intoxication. For example, approximately 40.7% of sexual minority women reported past month alcohol use compared to 45.3% of heterosexual women, but which was not a statistically significant difference. However, heterosexual women were more likely to report ever blacking out from alcohol intoxication (39.1%) relative to sexual minority women (28.3%) (χ^2^(1) = 6.32, *p* < 0.01). 

### 3.4. Beliefs about Sexual Violence 

Overall, 43.7% of participants reported desirable responses (correctly disagreed) to all seven sexual violence belief items; heterosexual women correctly disagreed with an average of 5.8 statements (SD = 1.49) and sexual minority women correctly disagreed with an average of 6.3 statements (SD = 0.94). This difference was statistically significant (t(275.9) = 5.95, *p* < 0.001). There were significant differences for six of the seven items. Sexual minority women were more likely to disagree (81.6%) with the statement “Most sexual assaults are committed by strangers” relative to heterosexual women (70.8%) (t(210.3) = 2.95, *p* = 0.004). Sexual minority women were more likely to disagree (73.1%) with the statement “Most sexual assaults occur in dangerous places (e.g., dark alleys)” relative to heterosexual women (63.5%) (t(199.7) = 2.34, *p* = 0.021). Sexual minority women were more likely to disagree (95.7%) with the statement “If a person doesn’t want to be sexually assaulted, they shouldn’t dress provocatively or act in a flirtatious way” relative to heterosexual women (86.3%) (t(297.9) = 4.53, *p* < 0.001). Sexual minority women were more likely to disagree (99.3%) with the statement “If someone didn’t report it to the police right away, they probably were not really sexually assaulted” relative to heterosexual women (94.0%) (t(591.2) = 4.87, *p* < 0.001). Sexual minority women were more likely to disagree (90.1%) with the statement “Victims often make up lies about being sexually assaulted” relative to heterosexual women (76.8%) (t(245.1) = 4.54, *p* < 0.004). Sexual minority women were more likely to disagree (97.2%) with the statement “If a person decides to go with someone to their bedroom, that implies consent to have sex” relative to heterosexual women (91.7%) (t(289.9) = 3.21, *p* < 0.001).

### 3.5. Attitudes about Sexual Consent

Overall, 33.8% of participants reported desirable responses to all five sexual consent attitude items. Sexual minority women reported desirable responses to an average of 4.21 statements (SD = 0.91) and heterosexual women agreed with an average of 3.84 statements (SD = 1.07), and this difference was statistically significant (t(212.2) = 4.32, *p* < 0.001). There were significant differences in four items. Sexual minority women, relative to heterosexual women, were more likely to correctly agree (97.2%) with the statement “It is the responsibility of both partners to make sure consent is established before sexual activity begins” (90.0%) (t(297.9) = 4.53, *p* < 0.001). Sexual minority women were more likely to correctly disagree (75.9%) with the statement “If consent is asked for at the beginning of a sexual encounter, it is not necessary to ask for consent at every step of the sexual interaction” relative to heterosexual women (61.7%) (t(205.8) = 3.56, *p* < 0.001). Sexual minority women were more likely to correctly disagree (62.4%) with the statement “Obtaining sexual consent is more important in a new relationship than in a long-term relationship” relative to heterosexual women (52.2%) (t(193.6) = 2.31, *p* = 0.022). Sexual minority women were more likely to correctly disagree (95.0%) with the statement “Sexual intercourse is the only sexual activity that requires explicit verbal consent” relative to heterosexual women (90.3%) (t(237.5) = 2.26, *p* = 0.025).

### 3.6. Predicting Behavioral Outcomes over Time

We conducted a series of logistic regression analyses examining predictors of self-reported sexual violence and substance use outcomes at the second time point. A logistic regression analysis examined sexual minority status and illicit drug use as predictors of sexual harassment victimization at Time 2, controlling for Time 1 sexual harassment victimization and for racial minority status—an analytic strategy that yields results that can be interpreted as change over time through the analysis of partial variance (e.g., identifying factors that predict increases in victimization during the first year of college in this sample). As shown in Table 4, findings indicated that bisexual women were 2.67 times more likely (OR = 2.67, 95% CI: 1.38, 5.16) to report new sexual harassment victimization relative to heterosexual women over this time period, and illicit drug use was associated with a 1.72 times increased risk (OR = 1.72, 95% CI: 1.15, 2.56) for sexual harassment victimization.

## 4. Discussion

A well-established body of research has shown that college students are at risk of sexual violence and substance abuse, but research on these issues among sexual minority youths, who may be especially vulnerable, has only recently begun to appear in the literature. Additionally, many national epidemiologic surveys have only recently begun to collect data on sexual orientation and gender identity. For example, the National Survey on Drug Use and Health (NSDUH) did not introduce a question asking about sexual orientation until 2015 [24]. Thus, it is important for researchers to collect information on sexual minority status to better understand behavioral and mental health disparities among sexual minority students and their needs for services and support. It is also important to examine risk behaviors among diverse women during the first year of university. This is often a period of new demands, challenges, freedoms, and social opportunities, some that significantly increase the risk for sexual violence and substance use. While previous research has shown that college women are at heightened risk for sexual violence, few studies have examined the confluence of victimization and perpetration, sexual orientation, and prevalent forms of alcohol and drug misuse influence outcomes. In this study of first-year college women who identified as heterosexual, bisexual, lesbian, asexual or questioning, rates of sexual violence victimization, perpetration, and substance use were examined. Findings indicated that victimization involving sexual harassment and sexual acts without consent were significantly higher among sexual minority women relative to heterosexual women, with bisexual women most likely to report these outcomes. 

Findings from the present study indicated that there are substantial differences between heterosexual women and women who are bisexual, lesbian, and asexual or questioning. Rates of victimization involving sexual harassment and sexual acts without consent were higher among sexual minority women relative to heterosexual women. Sexual minority women in their first year of college, particularly bisexual women, were significantly more likely to report sexual harassment (lifetime, past year, and past month) and sexual activity without consent (lifetime and past year) compared to first-year heterosexual university women. In the present study, more than one in four (28.0%) heterosexual university women reported experiencing sexual activity without consent during their lifetime (a number consistent with previous research [1]). Notably, more than one-third of lesbian (36.0%) and over half of bisexual (54.3%) university women in the present study reported this experience. In addition, experience with sexual harassment was very common in the present study, with more than two-thirds of heterosexual women (68.8%) and more than three-quarters of lesbian (88.0%) and bisexual women (85.1%) experiencing sexual harassment during their lifetime. A serious public health concern is that a third (32.8%) of heterosexual, 44% of lesbian, and over half (52.1%) of bisexual first-year university women experienced sexual harassment in the past month. Sexual minority women in their first year of college, particularly bisexual women, were significantly more likely to report lifetime sexual harassment perpetration when compared to first-year heterosexual university women. Furthermore, sexual minority women reported more frequent cigarette smoking, marijuana use and intoxication, use of club drugs, and overall illicit drug use relative to their heterosexual counterparts. 

A consistent finding across outcomes was that bisexual women were most at-risk for sexual violence, including both victimization and perpetration, as well as more deleterious substance use outcomes. Furthermore, we found that over the course of the first year, bisexual women and women who used illicit substances were most likely to report new incidents of sexual violence victimization. Overall, being bisexual was consistently associated with increased risk across multiple behavioral outcomes, indicating that emerging adult women who are bisexual are especially vulnerable during the first year of university—a critical year of transition. These findings are consistent with previous research in which bisexual university women are at greater risk of sexual assault compared to their heterosexual and gay/lesbian counterparts, regardless of gender [25,26]. Bisexual women tend to report more risk factors for sexual violence than heterosexual women, including earlier age at first sexual intercourse, greater number of sex partners, and child sexual abuse [6]. “Hooking-up”, having a greater number of partners, and risky sexual behaviors have also been associated with sexual assault among university women, including those who are bisexual [6]. However, an important consideration that was not assessed in the present study is the gender of the partners these women are being victimized by and it is unclear whether bisexual women in the sample were victimized by male or female partners. Nevertheless, previous studies have shown that bisexual women are more likely to report emotional and psychological violence and intimate stalking by male partners [27]. Moreover, negative attitudes about bisexuality and perceived or actual infidelity have been associated with intimate partner violence against bisexual women by their male partners [28]. Thus, future research should explore university students’ attitudes and beliefs about bisexuality and the extent to which negative attitudes and beliefs are associated with sexual violence against bisexual university women. Results from such efforts could be vital to inform sexual assault prevention efforts.

Our findings on substance use indicated that sexual minority women reported more frequent cigarette smoking, marijuana use and intoxication, use of club drugs, and overall illicit drug use relative to heterosexual women. Consistent with other findings of this study, bisexual women were most at risk for problematic substance use. These findings are also consistent with previous research that has found that lesbian and bisexual university women report more frequent substance use relative to their heterosexual counterparts [29,30]. In the university setting, bisexual women report the greatest risk for alcohol, tobacco, and other substance use compared to both lesbian and heterosexual university women [31,32]. However, our findings did not show consistent patterns of elevated alcohol use among sexual minority women. There were no significant differences across groups in terms of lifetime, past year, or past month alcohol use or alcohol intoxication. Indeed, heterosexual women were more likely to report ever blacking out from alcohol intoxication relative to sexual minority women. This finding suggests that elevated rates of sexual violence among sexual minority women were not primarily due to alcohol consumption. Instead, we found that rates of illicit drug use were higher among sexual minority women, and both bisexual status and illicit drug use were independent predictors of reporting sexual violence victimization over the course of the first year of university. 

This investigation also found that sexual minority status was associated with a greater likelihood of sexual harassment perpetration among first-year university women, with roughly 20% of lesbians and 25% of bisexual women reporting sexual harassment perpetration during their lifetime compared with 13.1% of heterosexual women. However, it is important to note that no differences were reported in more recent sexual harassment perpetration among these women (in the past year or the past month) and that perpetration rates were low among all university women. These findings are consistent with previous research in which sexual minority youths report greater sexual violence victimization and sexual harassment perpetration, and that most youths, including sexual minority youths, decrease sexual harassment perpetration over time [33]. This is likely because young people during their adolescent years learn that such behavior is inappropriate through educational programs and/or through their own experiences with sexual harassment and violence. This may also explain why first-year sexual minority women reported more accurate beliefs about sexual violence and consent, reinforcing the importance of sexual violence prevention education that is inclusive of sexual minority youths.

The present study has several strengths and limitations. Strengths include the geographically dispersed sample of first-year women attending 14 universities across the United States and the comprehensive longitudinal assessment of risk behaviors and relevant risk and protective factors. Limitations of the study are the possibility of underreporting of sensitive behaviors, small sample sizes for some sexual minority groups, and the lack of information on the gender of sexual partners and participants’ gender assigned at birth. Because of this, future studies should include larger sample sizes to confirm the present findings and examine these variables among diverse participants, including transgender university students. Furthermore, sampling methods should include focused strategies to include students from lesbian, gay, bisexual, transgender, and questioning (LGBTQ) clubs and alliance organizations, where there will be a higher concentration and diversity of sexual minority students than in the general student body. An additional limitation is that the Cronbach alpha for the sexual consent attitude scale was relatively low, although considered acceptable for exploratory research [34]. Other limitations include the potential for inaccurate recall of events that occurred in the past, and the possibility that additional confounding variables were not measured and controlled for in the data analysis. 

## 5. Conclusions

Overall, these findings indicate that first-year sexual minority university women are at substantially greater risk of experiencing sexual violence than first-year heterosexual university women. Since the first year of college is considered the riskiest time for women to experience sexual violence, it is important that sexual assault prevention efforts address anti-LGBTQ attitudes and beliefs and that treatment services for sexual assault are inclusive of sexual minority women, particularly bisexual women. Moreover, because sexual minority women are more likely than heterosexual women to enter college having already experienced sexual violence, LGBTQ-inclusive prevention programs and treatment services must be offered to students at much younger ages. 

Sexual minority adolescents and young adults are at elevated risk of numerous negative behavioral and mental health outcomes, including problems with depression, anxiety, and suicidal ideation and behavior, in addition to sexual violence and substance use [35]. These health disparities, consistent with minority stress theory, may result from greater social stress, societal stigma, discrimination, and inequality [36]. Therefore, public health initiatives are needed to address these systemic issues, and these efforts should include prevention programs that aim to enhance overall resilience through social and emotional skills training for sexual minority youths, both during the college years and in the years of secondary school. 

## Figures and Tables

**Table 1:** Sexual violence outcomes by sexual minority status.

	Heterosexual (a)	Sexual Minority (b)	(a) vs. (b)	Lesbian (c)	Bisexual (d)	Asexual /Questioning (e)	(a) (c)–(e)
	*n* = 833	*n* = 141		*n* = 25	*n* = 94	*n* = 22	
Sexual Harassment Victimization							
Lifetime	68.8%	83.7%	χ^2^ = 12.99 ***	88.0%	85.1%	72.7%	F = 4.92 **; (d) > (a) **
Past Year	59.9%	73.8%	χ^2^ = 9.82 ***	76.0%	77.7%	54.6%	F = 4.68 **; (d) > (a) **
Past Month	32.5%	46.8%	χ^2^ = 10.86 ***	44.0%	52.1%	27.3%	F = 5.34 ***; (d) > (a) **
Sexual Activity without Consent Victimization							
Lifetime	28.0%	48.2%	χ^2^ = 23.17 ***	36.0%	54.3%	36.4%	F = 9.56 ***; (d) > (a) ***
Past Year	14.3%	23.4%	χ^2^ = 7.61 **	8.0%	28.7%	18.2%	F = 4.92 **; (d) > (a) ***
Past Month	5.2%	6.4%	χ^2^ = 0.36	4.0%	8.5%	0.0%	F = 1.08
Sexual Harassment Perpetration							
Lifetime	13.1%	22.2%	χ^2^ = 7.76 **	20.0%	25.5%	9.1%	F = 3.96 **; (d) > (a) **
Past Year	9.4%	12.8%	χ^2^ = 1.57	12.0%	14.9%	4.6%	F = 1.25
Past Month	4.4%	5.6%	χ^2^ = 0.42	0.0%	8.5%	0.0%	F = 1.86
Sexual Activity without Consent Perpetration							
Lifetime	1.1%	0.0%	χ^2^ = 1.54	0.0%	0.0%	0.0%	F = 0.51
Past Year	0.5%	0.0%	χ^2^ = 0.68	0.0%	0.0%	0.0%	F = 0.23
Past Month	0.2%	0.0%	χ^2^ = 0.34	0.0%	0.0%	0.0%	F = 0.11

Note: ** *p* < 0.01, *** *p* < 0.001.

**Table 2:** Substance use outcomes by sexual minority status.

	Heterosexual (a)	Sexual Minority (b)	(a) vs. (b)	Lesbian (c)	Bisexual (d)	Asexual /Questioning (e)	(a) (c)–(e)
	*n* = 833	*n* = 141		*n* = 25	*n* = 94	*n* = 22	
Substance Use							
Lifetime	72.4%	74.5%	χ^2^ = 0.26	76.0%	74.5%	72.7%	F = 0.11
Past Year	68.5%	68.1%	χ^2^ = 0.01	72.0%	71.3%	50.0%	F = 1.32
Past Month	48.7%	48.9%	χ^2^ = 0.01	52.0%	52.1%	31.8%	F = 1.002
Prescription Drug Misuse							
Lifetime	4.6%	7.1%	χ^2^ = 1.65	8.0%	8.5%	0.0%	F = 1.49
Past Year	3.4%	5.0%	χ^2^ = 0.90	7.0%	5.3%	0.0%	F = 1.05
Past Month	1.6%	1.4%	χ^2^ = 0.02	0.0%	2.1%	0.0%	F = 0.32
Illicit Drug Use							
Lifetime	33.0%	44.0%	χ^2^ = 6.40 *	36.0%	48.9%	31.8%	F = 3.21 *; (d) > (a) **
Past Year	27.7%	36.9%	χ^2^ = 4.90 *	33.0%	38.3%	28.7%	F = 1.76
Past Month	17.3%	27.7%	χ^2^ = 8.50 **	28.0%	29.9%	18.2%	F = 3.38 *; (d) > (a) **

Note: * *p* < 0.05, ** *p* < 0.01.

**Table 3:** Alcohol use outcomes by sexual minority status.

	Heterosexual (a)	Sexual Minority (b)	(a) vs. (b)	Lesbian (c)	Bisexual (d)	Asexual /Questioning (e)	(a) (c)–(e)
	*n* = 833	*n* = 141		*n* = 25	*n* = 94	*n* = 22	
Alcohol Use							
Lifetime	69.5%	69.3%	χ^2^ = 0.01	75.0%	70.2%	59.1%	F = 0.50
Past Year	65.9%	62.9%	χ^2^ = 0.52	66.7%	67.0%	40.9%	F = 2.03
Past Month	45.3%	40.7%	χ^2^ = 1.12	45.8%	43.6%	22.7%	F = 1.52
Alcohol Intoxication							
Lifetime	51.5%	46.8%	χ^2^ = 1.100	43.5%	52.1%	27.3%	F = 1.88
Past Year	47.2%	43.0%	χ^2^ = 0.98	34.8%	48.9%	23.7%	F = 1.71
Past Month	31.7%	28.8%	χ^2^ = 0.60	21.7%	34.0%	13.6%	F = 1.56
5+ Drinks per Occasion	13.1%	6.4%	χ^2^ = 5.11 *	4.0%	8.5%	0.0%	F = 0.69
Blacked Out from Drinking	39.1%	28.3%	χ^2^ = 6.32 **	20.0%	32.9%	18.2%	F = 2.96 *

Note: * *p* < 0.05, ** *p* < 0.01.

**Table 4:** Multiple regression analysis of sexual harassment victimization at Time 2.

	B	S.E.	Wald χ^2^	OR	95% CI
Sexual Harassment Victimization at Time 1	2.13	0.19	131.99 ***	8.39	5.84, 12.05
Lesbian	0.04	0.70	0.01	1.05	0.26, 4.15
Bisexual	0.98	0.34	8.52 **	2.67	1.38, 5.16
Asexual/Questioning	0.66	0.58	1.28	1.93	0.62, 6.00
Racial Minority	−0.25	0.20	1.53	0.78	0.52, 1.16
Illicit Drug Use in Past Year	0.54	0.20	7.07 **	1.72	1.15, 2.56

Note: ** *p* < 0.01, *** *p* < 0.001; Heterosexual was the reference group for each sexual minority category.

## Data Availability

The raw data used in this study is available from the corresponding author upon reasonable request.
